# The Re-Emergence of Mpox: Old Illness, Modern Challenges

**DOI:** 10.3390/biomedicines12071457

**Published:** 2024-07-01

**Authors:** Mohammad Ali Zinnah, Md Bashir Uddin, Tanjila Hasan, Shobhan Das, Fahima Khatun, Md Hasibul Hasan, Ruenruetai Udonsom, Md Masudur Rahman, Hossam M. Ashour

**Affiliations:** 1 Department of Microbiology and Public Health, Bangabandhu Sheikh Mujibur Rahman Agricultural University, Gazipur 1706, Bangladesh; 2 Department of Medicine, Faculty of Veterinary, Animal and Biomedical Sciences, Sylhet Agricultural University, Sylhet 3100, Bangladesh; 3 Department of Medicine and Surgery, Faculty of Veterinary Medicine, Chattogram Veterinary and Animal Sciences University, Chattogram 4225, Bangladesh; 4 Jiann-Ping Hsu College of Public Health, Georgia Southern University, Statesboro, GA 30458, USA; 5 Department of Pathobiology, Bangabandhu Sheikh Mujibur Rahman Agricultural University, Gazipur 1706, Bangladesh; 6 Bangabandhu Sheikh Mujibur Rahman Science and Technology University, Gopalganj 8100, Bangladesh; 7 Department of Protozoology, Faculty of Tropical Medicine, Mahidol University, Bangkok 73170, Thailand; 8 Department of Pathology, Faculty of Veterinary, Animal and Biomedical Sciences, Sylhet Agricultural University, Sylhet 3100, Bangladesh; 9 ABEx Bio-Research Center, East Azampur, Dhaka 1230, Bangladesh; 10 Department of Integrative Biology, College of Arts and Sciences, University of South Florida, St. Petersburg, FL 33701, USA

**Keywords:** Mpox, MPXV, emergence, wild rodents, zoonosis

## Abstract

The Mpox virus (MPXV) is known to cause zoonotic disease in humans. The virus belongs to the genus Orthopoxvirus, of the family Poxviridae, and was first reported in monkeys in 1959 in Denmark and in humans in 1970 in the Congo. MPXV first appeared in the U.S. in 2003, re-emerged in 2017, and spread globally within a few years. Wild African rodents are thought to be the reservoir of MPXV. The exotic trade of animals and international travel can contribute to the spread of the Mpox virus. A phylogenetic analysis of MPXV revealed two distinct clades (Central African clade and West African clade). The smallpox vaccine shows cross-protection against MPXV infections in humans. Those who have not previously been exposed to Orthopoxvirus infections are more vulnerable to MPXV infections. Clinical manifestations in humans include fever, muscle pain, headache, and vesicle formation on the skin of infected individuals. Pathognomonic lesions include ballooning degenerations with Guarnieri-like inclusions in vesicular epithelial cells. Alterations in viral genome through genetic mutations might favor the re-emergence of a version of MPXV with enhanced virulence. As of November 2023, 92,783 cases and 171 deaths have been reported in 116 countries, representing a global public health concern. Here, we provide insights on the re-emergence of MPXV in humans. This review covers the origin, emergence, re-emergence, transmission, pathology, diagnosis, control measures, and immunomodulation of the virus, as well as clinical manifestations. Concerted efforts of health professionals and scientists are needed to prevent the disease and stop its transmission in vulnerable populations.

## 1. Introduction

Mpox (formerly known as monkeypox) is a transmissible disease that can impact humans and animals. The Mpox virus (MPXV) was first described in humans in the 1970s in the Democratic Republic of the Congo (DRC) causing a primarily endemic disease throughout the rainforests of Central and Western Africa with no reported outbreaks elsewhere [1]. In 2003, Mpox was reported in Wisconsin, USA [2] and was later reported in other countries outside Africa. This threat, if not contained, can potentially add to the economic losses the world has been facing since the advent of the COVID-19 pandemic era in 2019 [3]. Coinfections of MPXV and SARS-CoV-2 could enhance pathogenicity, infectivity, and/or response to vaccines in one or both cases [4]. Possible interactions between the two viruses could also trigger the emergence of new variants of SARS-CoV-2 with hosts having enhanced immune evasion capabilities [5]. Considering its characteristics and risks, MPXV has been assigned to the biosafety level 3 (BSL-3) category by the EU [6], and was similarly categorized in the Selected Agents and Toxin List in the U.S. [7]. 

Due to its generally reduced severity, Mpox cases suffer from underreporting and are more prone to poor case management [8]. The recent surge in case counts of Mpox is changing this. There is fear that MPXV might be the next emerging pathogen from the Poxviridae family after smallpox. Thus, prevention is both critical and timely. In this review, we summarize the latest publicly available information on the origin, evolution and emergence, transmission, pathology, diagnosis, and control of MPXV.

## 2. Outbreaks

Recent Mpox outbreaks around the world highlight the importance of improved universal health care systems. The unpredictable and widespread nature of MPXV indicates the shortcomings of current surveillance systems. Considering the complex impact of MPXV infections on pregnant women, children, and immunosuppressed individuals, these clusters of the population need to be included in the disease surveillance system. Based on the recent Mpox outbreaks as reported by the Centers for Disease Control and Prevention (CDC), the number of cases tends to be higher in individuals aged 26 to 40 [9]. The WHO closely monitors outbreaks and takes action by sharing information and coordinating with member states and partners. From 1 January 2022 to 30 November 2023, a total of 92,783 Mpox cases and 171 deaths have been identified and reported to the WHO from 116 countries/territories/areas in 6 WHO regions [10]. 

## 3. Origin and Emergence

In 1959, MPXV was reported as the etiologic agent of disease in a group of cynomolgus monkeys (*Macaca fascicularis*) at a research station in Denmark [11]. In the 1970s, the Mpox virus was reported to cause a smallpox-like illness in humans in the Democratic Republic of the Congo [12]. Since the 1970s, the virus has been endemic in Africa until 2003, when a human case was identified in the U.S. as a result of an infection from prairie dogs (*Cynomys* spp.). The dogs were apparently infected after coming into contact with exotic mammals and African rodents (*Funisciurus* spp., *Heliosciurus* spp., *Cricetomys* spp., *Atherurus* spp., *Graphiurus* spp., and *Hybomys* spp.) imported from Ghana [13]. Subsequently, sporadic occurrences of the disease were reported in other non-African countries. Recent rapid dissemination has made this disease a current global concern.

Monkeys living in the rainforests of Africa, presumed to have been naturally infected with MPXV, were thought to be the source of infection to humans through mingling with local villagers [14]. Mpox infections in humans lead to lesions that are very similar to lesions caused by smallpox, and eradication programs against smallpox could help fight Mpox [15]. It is noteworthy that before the 1970s, the virus was thought to be circulating only among captive primates [16]. The cessation of smallpox vaccination programs in the Democratic Republic of the Congo (formerly called the Republic of Zaire) in 1980 potentially contributed to the increased number of human Mpox cases in West and Central Africa [14]. 

MPXV is a serious emerging and re-emerging zoonotic pathogen, which has a wide host range [17]. Rodents might act as potential reservoirs of MPXV due to their close host–pathogen relationship with other members of the Orthopoxvirus [18].

## 4. Classification and Clades

**Figure 1:**
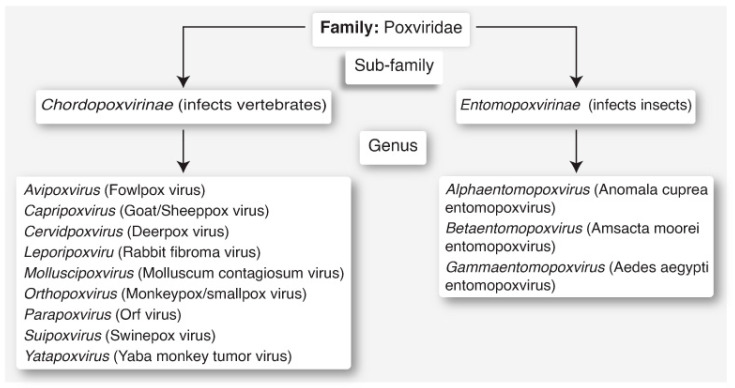
Classification of poxviruses, of the family Poxviridae. The Mpox virus belongs to the sub-family *Chordopoxvirinae*, of the genus *Orthopoxvirus*.

The causative agent of Mpox, MPXV, is a member of the genus Orthopoxvirus, of the family Poxviridae [19]. The genus Orthopoxvirus includes a total of twelve viruses (Figure 1) such as the cowpox virus (CPXV), variola virus (VARV), and vaccinia virus (VACV), which can also cause human diseases [20]. MPXV is a brick-shaped enveloped virus with linear double-stranded DNA (dsDNA), a genome size of 170–250 kb, a measurement of 200–250 nm, and a mechanism of entry into host cells that involves binding to glycosaminoglycans [21]. It is likely that apoptotic mimicry allows viral entry into host cells [22]. This is followed by viral replication within the host cells.

A molecular analysis of the genetic materials of MPXV reveals two distinct clades, which represent different levels of pathogenicity of the virus: clade I (formerly the Congo Basin clade or the Central African clade) and clade II (the West African clade) [23]. The case fatality rate (CFR) of clade I is 10.6% (associated with more severe infections in humans and non-human primates), whereas clade II’s CFR is only 3.6% [24,25]. Thus, genetic differences between the clades might be responsible for the varying pathogenicity, virulence, and transmission patterns. Genes that may be responsible for the increased virulence of clade I include the host range protein (D10L), complement inhibitor (D14L), apoptotic regulator (B10R), IL-1β binding protein (B14R), and serine protease inhibitor-like protein (B19R) [23,26]. It is noteworthy that clade II lacks D14L, making this gene a particularly interesting gene for further studies, as it may be primarily responsible for the higher virulence in clade I [26,27]. 

## 5. Genetic Factors

Gene gain and loss through genetic mutations in Orthopoxvirus may increase the adaptation capabilities of viruses to their alternative hosts and potentially enhance viral virulence [28]. The adaptation of viruses to their hosts and viral mutations can also occur through nucleotide changes and through host factors such as DNA- and RNA-editing enzymes (including host APOBEC cytosine deaminases) [29]. 

**Figure 2:**
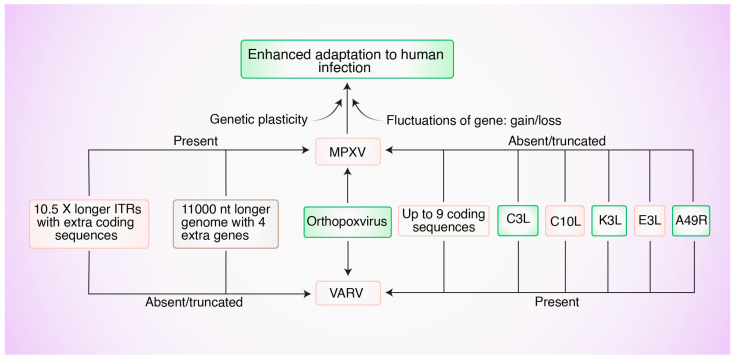
Distinctions between the Mpox virus (MPXV; Zaire-96 strain) and the variola virus (VARV; Kuwait-1967 strain) of the genus Orthopoxvirus. C3L, C10L K3L, E3L, and A49R loci are present in VARV but absent in MPXV. Moreover, MPXV has 10.5× longer ITRs with extra coding sequences and 11,000 longer nucleotide genomes with 4 extra genes. ITR: inverted terminal repeats; D14L: ortholog of the C3L gene; C3L: virulence-associated gene; C10L: gene coding IL-1B antagonist protein; K3L: interferon resistance gene; E3L: interferon resistance gene; A49R: gene coding phospho-transferase protein.

Variola and Mpox are two Orthopoxviruses under the sub-family Chrodopoxvirus. In addition to possessing the proposed minimum essential genome of Chrodopoxviruses, both variola and Mpox possess genes that have been linked to host specificity, immune regulation, and subcellular transport. As shown in Figure 2, MPXV and VARV appeared to have evolved in an independent manner from one Orthopoxvirus ancestor [30]. Compared to the genome of the Kuwait-1967 strain of variola, the MPXV genome (Zaire-96 strain) has 4 more genes, is 11,000 nucleotides longer, has 10.5-times longer inverted terminal repeats (ITRs), and has extra coding sequences within the ITRs (Figure 2) [31]. Conversely, the genome of variola does not have any extra coding sequences (Figure 2) [31]. Although the genome of variola is one of the smallest among Orthopoxviruses, it has up to nine defined coding sequences, which are absent in the MPXV genome (Figure 2) [27]. The presence or absence of genes might be associated with enhanced virulence in human hosts. This necessitates further experimentation.

As with other DNA viruses, genetic mutations in MPXV are less frequent than mutations in RNA viruses [32]. However, MPXV has a suggested mutation rate that is estimated to be 10-fold higher than what would be expected from a standard identical DNA virus [33]. It is noteworthy that MPXV isolates from the 2022 outbreak had 40 similar but unique mutations that were not shared with the closest variant [33]. Whereas some viral mutations, induced through their interaction with the host’s immune system, may not impact the virus or have minimal impact, others can be deleterious to the virus [34]. 

The enzyme, apolipoprotein B mRNA-editing catalytic polypeptide-like 3 (APOBEC3), can cause cytosine deamination into uracil in viral genomes [35]. The AT content of the MPXV genome is two-fold higher than the GC content, indicating a role for the APOBEC3 enzyme in viral mutations [36]. In fact, recent MPXV genome analyses showed that APOBEC3-editing enzymes can be responsible for up to 90% of new nucleotide alterations [36]. 

Genetic recombination can also cause viruses from the *Poxviridae* family to acquire or lose genes [37]. Thus, genetic and phenotypic diversity among Orthopoxviruses can be partially attributed to genetic recombination events [38]. High-frequency recombination has been reported in poxviruses while replicating within infected host cells [39]. Inter-species recombination has been proven among and between Orthopoxviruses including cowpox and ectromelia viruses [40]. New mechanisms of poxvirus adaptations need to be explored further to fully understand potentially complex genome dynamics in poxviruses including MPXV [41]. In light of reports of coinfection of MPXV with the Varicella-Zoster virus (VZV) [8,42] and HIV [43], the possibility of recombination between MPXV and other Orthopoxviruses during coinfection requires intense investigation. 

## 6. Hosts and Transmission

There is still uncertainty regarding the natural host of MPXV. However, MPXV can cause diseases in a broad range of mammalian hosts. The virus has been previously isolated from the rope squirrel (*Funisciurus anerythrus*) in the DRC [44] and the sooty mangabey (*Cercocebus atys*) in the Ivory Coast [45]. It can infect rodents in Africa including prairie dogs (*Cynomys* spp.) and Gambian pouched rats *(Cricetomys gambianus)* [46]. In addition, *Funisciurus* spp. (rope squirrels), *Graphiurus lorraineus* (African dormouse), *Cricetomys emini* (African giant pouched rats), *Heliosciurus* spp. (sun squirrels), *Oenomys hypoxanthus* (rufous-nosed rats), and *Petrodromus tetradactylus* (elephant shrew) have been found to exhibit seropositivity to MPXV [47]. Furthermore, the wild boar (*Sus scrofa)* and the rhesus macaque (*Macaca mulatta*) could be vulnerable to MPXV infection [48]. 

**Figure 3:**
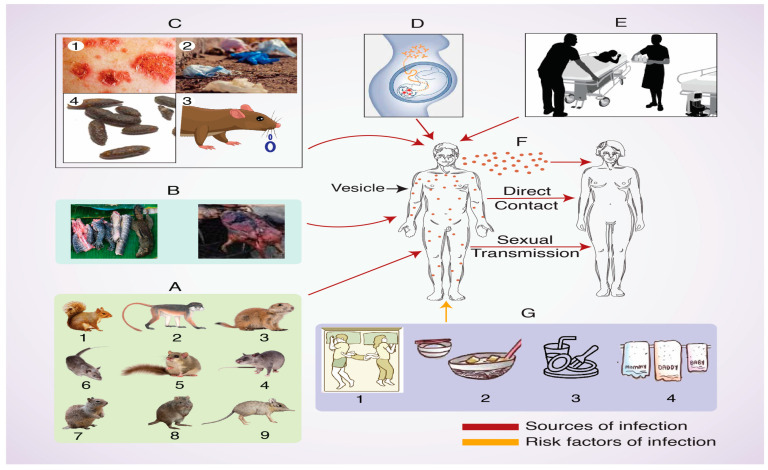
Transmission of Mpox. Schematic illustration to show the different routes of transmission. In (**A**), the numbers correspond to the following animals: 1. rope squirrel; 2. sooty mangabey; 3. prairie dog; 4. Gambian pouched rat; 5. African dormice rodent; 6. African giant pouched rat; 7. sun squirrel; 8. rufous-nosed rat; and 9. elephant shrew. (**B**) represents bush meat. In (**C**), the numbers correspond to the following: 1. skin crust; 2. patients’ used materials; 3. contaminated saliva; and 4. fecal materials. (**D**) represents transplacental transmission. (**E**) reflects hospital-borne infection. (**F**) shows transmission by respiratory droplets and direct contact. (**G**) shows sharing of 1. bed; 2. food; 3. a glass and other utensils; and 4. hand towels.

Transmission of MPXV can occur via contaminated contact with saliva, respiratory droplets, exudates of lesions, crust, patients’ used materials, and feces [49,50]. Risk factors that facilitate human transmission of the disease include bed and room sharing, dish and utensil sharing, and living in the same household [51]. Risk factors for animal-to-human transmission include staying in close proximity to infected animals or to areas where infected animals may be present [52]. As a hospital-borne infection [53,54], Mpox can occur through venereal transmission from infected patients with genital lesions to healthy individuals [43]. Finally, transplacental transmission during pregnancy could result in fetal death [55] (Figure 3).

## 7. Pathogenesis

Pathogenesis includes various steps such as viral entry, fusion, replication, and viral release from the host cell. The routes of entry of MPXV into host cells include sexual routes, intradermal routes, oropharynx, and nasopharynx. Once inside, the virus replicates and causes primary viremia and secondary viremia, resulting in the involvement of local lymph nodes and systemic circulation and culminating in skin and mucosal infection. The infection can also spread to other organs including the lungs, eyes, and gastrointestinal tract. The two infectious forms of the virus include single membrane-bound intracellular mature virions (MV) and triple membrane-bound extracellular enveloped virions (EV) [56].

Unlike other DNA viruses that replicate in the host cell nucleus, MPXV and other double-stranded DNA Orthopoxviruses replicate in the cellular cytoplasm. Whereas enzymes required for viral replication and structural protein synthesis are encoded by highly conserved genes positioned at the central part of the viral genome, enzymes required for viral interaction with the host are encoded by less conserved genes located at the terminal part of the viral genome [57]. Proteins deemed necessary for viral DNA replication, transcription, assembly, and release are typically encoded by genes in the more diverse terminal regions [57].

## 8. Clinical Hallmarks

**Figure 4:**
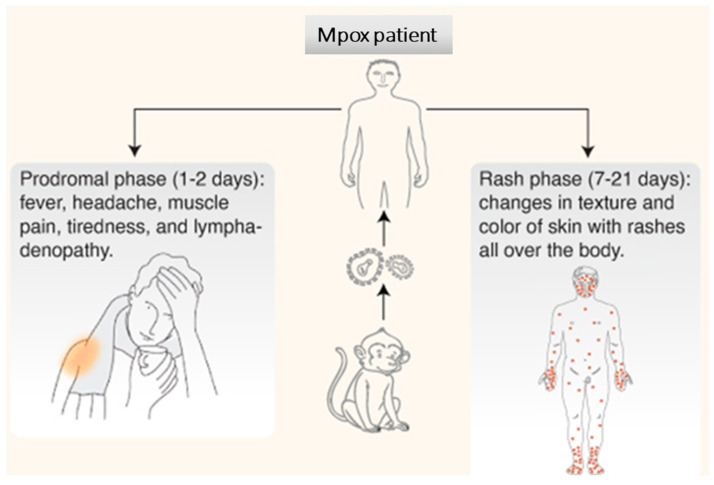
Clinical manifestations of Mpox infections in humans.

The clinical signs of Mpox infections are generally similar but less severe than symptoms of smallpox infections. The incubation period usually ranges from one to two weeks. It is recommended that patients are monitored for three weeks. The clinical history of patients usually involves contact with infected animals or humans. As in Figure 4, the first of the two phases of MPXV infection is the prodromal phase. This phase lasts for 1–2 days and is characterized by fever, severe headache, muscle ache, tiredness, and lymphadenopathy. Lymphadenopathy can be a distinguishing feature for Mpox infections as compared to other viral rash diseases. The second phase is the rash phase, which can last for two weeks or more and is characterized by abnormal changes in the texture and color of the skin throughout the body. Patients stay contagious throughout the period from when symptoms start until the rashes completely heal. Rashes are typically concentrated on the face but also include other parts of the body such as the palms of the hands, soles of the feet, oral mucosa, and, to a lesser extent, on the genitalia and conjunctiva. Rashes can persist for weeks and progress in stages including macules, papules, vesicles, and pustules, followed by scabs and crusts, which are ultimately shed as part of the resolution stage [58]. The vast majority of human Mpox patients develop lymphadenopathy in the groin and other areas of the body. Complications include secondary bacterial infections, bronchial pneumonia, respiratory distress, encephalitis, corneal infection, vision loss, and dehydration through vomiting and diarrhea [58,59]. Typically, Mpox is self-limiting as it rarely results in death. The severity of the disease depends on many factors, including the health status of patients and the degree of exposure to the virus.

## 9. Pathological Features

In prairie dogs, the disease can lead to the eyelids excreting a yellowish mucoid discharge, ulceration in the middle of the tongue, red–brown patchy zones in pulmonary parenchyma, and red livers with several scattered and tanned spotted areas [60]. Lesions in humans are initially found around the genitalia but can then spread to other areas of the body including the oral mucosa, conjunctiva, face, palms, and toes [61,62]. Cutaneous lesions include whitish papules with significant edema and umbilication, leading to crusts and erosions [61].

The histopathology of the eyelids in prairie dogs revealed ulcerated lesions in the conjunctiva, which contain necrotic debris and pyknotic epithelial cells and are surrounded by swollen columnar epithelial cells with Guarnieri-like inclusions [60]. Examining the submucosa revealed inflammatory cell infiltration along with necrosis and edema [60]. The hallmarks of the disease in skin and palpebral conjunctiva include the ballooning degeneration of the epithelial cells, acantholysis, and cellular necrosis [60]. The airways of the lung show necrosis and appear to be infiltrated with neutrophils and histiocytes in both the lumen and epithelium [60]. In addition to the inflamed bronchiolar walls, the surrounding alveoli exhibit edema, necrosis, and the marked infiltration of macrophages [60]. In humans, the histology of the vesicular and pustular stages is well-documented. The vesicular stage comprises bulla with multinucleated keratinocytes and the ballooning degeneration of keratinocytes with a mixed inflammatory infiltrate [62]. The pustular stage consists of bulla with mostly non-viable keratinocytes, a few multinucleated keratinocytes, rare keratinocytes displaying an eosinophilic ‘ground glass’ appearance in the center of the nucleus, and the inflammatory infiltration of lymphocytes, neutrophils, and eosinophils [62]. 

## 10. Host Immune Responses to MPXV Infections

Poxviruses use different mechanisms to try to evade the host’s immune response [63]. Natural killer (NK) cells, known to kill virus-infected cells through perforin, granzymes, and cell–cell contact, can secrete cytokines that promote effective immune responses. Interactions between receptors on NK cells and major histocompatibility complex-1 (MHC-1) molecules can dictate whether NK cells are activated. Humoral immune responses appear to play significant roles in protecting against MPXV infections [64]. The role of NK cells in response to an MPXV infection has been documented in rhesus macaques [65]. MPXV possesses the ability to infect primary human monocytes while evading specific T cell responses by blocking antiviral CD4^+^ and CD8^+^ T cell activation and inhibiting proinflammatory cytokines (such as IFN-γ and TNF-α) [66]. Poxviruses are thought to have different mechanisms to avoid the host’s immune response [67]. In order to fully understand the virus–host relationship between zoonotic DNA viruses (such as MPXV) and their hosts, a new immune-centric understanding needs to be developed [68]. Mechanisms of the innate and adaptive immune responses against MPXV infections need to be further explored.

## 11. Diagnosis

Challenges for differentially diagnosing Mpox include similarities of clinical signs to signs of smallpox infections, and to infections of chickenpox, measles, bacterial skin infection, medication allergies, and syphilis. Quick and sensitive diagnostic tests are needed to control the spread of Mpox. Sero-diagnostic methods using antibodies that show cross-reactivity to other members of the Orthopoxvirus may not be very helpful for a definite diagnosis of Mpox. The discovery of more specific antibodies, such as the 69-126-3-7 monoclonal antibody (mAb) that binds to MPXV A29, is a step in the right direction [69]. Diagnostic methods for Mpox include PCR, electron microscopy, cultures from rash specimens, immunohistochemistry (for detection of viral antigens), and serological detection [59]. 

In order to detect the DNA of MPXV in clinical samples, RT-PCR should target the gene of the extracellular-envelope protein (B6R), DNA polymerase gene (E9L), DNA dependent RNA polymerase subunit 18 (rpo18), and F3L [70]. A highly specific and sensitive assay called recombinase polymerase amplification (RPA) was developed for the rapid detection of the G2R gene of MPXV [71]. The development of newer assays based on PCR and genome sequencing could revolutionize the diagnosis of Mpox. 

## 12. Immunoprophylaxis and Therapy

Immunoprophylaxis conferred by vaccination can be safer and more effective. As an example, 4pox is a DNA vaccine that was developed using gene-based technology and was given this name because it targets four Orthopoxvirus antigens [72]. These antigens are L1 and A27 immunogens for mature virions of Orthopoxvirus, and A33 and B5 immunogens for enveloped virions of Orthopoxvirus [72]. This vaccine, and other similar vaccines, can prevent the shedding of viral particles in the vaccinated host, causing a decrease in the severity of the viral infection [72]. Immunization against smallpox can also confer protection against MPXV [59]. In fact, the smallpox vaccine, ACAM2000, was at one point recommended by the Centers for Disease Control and Prevention (CDC) to better manage the symptoms of MPXV after the infection has already occurred [2,59]. However, later safety concerns led to stopping the use of ACAM2000 [59]. 

The FDA-approved MPXV/smallpox vaccine, JYNNEOS, is a third-generation vaccine that is based on live, attenuated modified vaccinia Ankara (MVA) and is not known to pose any major safety concerns or cutaneous reactions [73]. The dosing regimen is two doses that are four weeks apart [73]. Booster doses are recommended for at-risk individuals who have continual viral exposure [73]. Participant-based active surveillance has recently been conducted in Italy to investigate adverse events following immunization (AEFIs) with the Imvanex/JYNNEOS vaccine [74]. The findings prove that the Mpox vaccine has a high tolerability profile, which particularly applies for common short-term systemic AEFIs [74]. The incidence and severity of local AEFIs were high, indicating the need to closely monitor these events following the intradermal administration of the vaccine [74]. 

Immunoprophylactic treatment with the monoclonal antibodies c7D11 (human–mouse chimera that targets the mature form of the virus) and c8A (human–chimpanzee chimera that targets the enveloped form of the virus) conferred protection against a lethal dose of MPXV [75]. Immunoprophylactic and therapeutic treatments with a recombinant vaccinia virus immunoglobulin (rVIG) were well-tolerated and led to high neutralizing activities against Orthopoxviruses in vitro and in vivo [76]. The immunotherapeutic effect was observed when a single intraperitoneal injection of rVIG was administered up to 14 days before challenge [76]. The therapeutic effect was observed when rVIG was administered up to 6 days post challenge [76]. 

Tecovirimat (also known as TPOXX and formerly known as ST-246) is an FDA-approved antiviral agent for smallpox [77]. Although the drug showed efficacy as a post-exposure prophylactic against MPXV, there is not enough research on its efficacy in human cases of MPXV [70,77]. The mode of action involves preventing the release of intracellular viruses from host cells [70]. Viral resistance to Tecovirimat has developed as a result of F13L gene mutations [77]. This resistance could be overcome by PAV-164, a methylene blue derivative that can inhibit MPXV replication [78]. 

## 13. Future Recommendations

Even though Mpox infections are less severe than smallpox infections, MPXV has the potential to become highly pathogenic due to its ability to mutate and undergo the genetic recombination characteristic of the *Poxviridae* family [37,39]. More data are needed about the recombination of MPXV with Orthopoxviruses during coinfection or superinfection. There are also reports about the coinfection of MPXV with the Varicella-Zoster virus (VZV) [8,42] and HIV [43]. Future endeavors should cover the consequences of coinfections and superinfections in patients with MPXV.

In spite of educated guesses and speculations, the animal reservoir is yet to be identified [79,80]. Expanding research in the areas of host and tissue tropism in the context of MPXV infections can enable us to have a better understanding of the spread of the virus and the host’s immune response [70]. 

The re-emergence of MPXV infections could be considered a consequence of multiple factors. One factor is the decline in herd immunity in the population as a result of the cessation of the smallpox vaccination. Another main factor is the more frequent interactions between humans and potential MPXV reservoirs as a result of deforestation, urbanization, and the handling and consumption of certain food products, such as bush meat ingestion. This might have resulted in the creation of new ecological and immunological niches for the spread of MPXV. The previous factors and recent outbreaks highlight the urgent need for consistent surveillance and the advancement of new immunoprophylactic and therapeutic strategies for MPXV. This should make us more prepared to handle potential future pandemics.
